# Multitasking Proteins and Their Involvement in Pathogenesis

**DOI:** 10.3390/cells12111460

**Published:** 2023-05-24

**Authors:** Agnieszka Gizak

**Affiliations:** Department of Molecular Physiology and Neurobiology, University of Wrocław, ul. Sienkiewicza 21, 50-335 Wroclaw, Poland; agnieszka.gizak@uwr.edu.pl

The “one protein, one function” paradigm, similar to the “one gene, one enzyme” hypothesis that dominated our thinking for a long time, has proven to be too simplistic. The identification of multitasking proteins has revealed a whole new level of cellular complexity and forced us to revise our views on the regulation of physiological and pathological processes in the cell. During evolution, proteins that have acquired additional abilities allow the cell to extend its spectrum of functionalities using a relatively limited set of genes. Since these non-canonical functions of proteins often contribute to development of human pathologies, understanding the phenomenon of multitasking could lead to development of new therapeutic interventions for diseases.

The scientific literature distinguishes between four manifestations of the multifunctionality phenomenon: pleiotropy, multidomain proteins, promiscuity, and moonlighting [1].

Pleiotropy is the phenomenon in which a single molecular function is involved in multiple biological processes [2]. Multidomain proteins comprise a subset of multifunctional proteins that are composed of “recurrent protein fragments with distinct structure, function and/or evolutionary history” [3]. Promiscuity refers to multiple catalytic activities of a protein or its binding to multiple ligands [1]. Moonlighting describes the widespread—as it turned out—phenomenon in which a protein performs multiple mechanistically independent functions without partitioning these functions into different polypeptide chains [4]. Importantly, all these types of multitasking could, at least in theory, manifest in a single protein.

The purpose of this Special Issue was to summarize the latest discoveries in this area of research.

In the paper entitled “Role of Moonlighting Proteins in Disease: Analyzing the Contribution of Canonical and Moonlighting Functions in Disease Progression” [5], Huerta et al. present the results of their studies on the link between the functions of moonlighting proteins and diseases. The authors point out that almost 80% of moonlighting proteins are implicated in human diseases, and over a quarter are involved in the pathogenicity of microorganisms. They emphasize that the activation of proteins’ moonlighting functions may result in disease promotion, in addition to restoring cellular homeostasis (or, at least, reducing the pathology-induced damage). Moreover, numerous moonlighting proteins may play distinct roles in different stages of disease development. This entails additional challenges for studies of pathogenesis, as well as therapeutic targeting.

Glycogen synthase kinase-3 (GSK-3) is a classic example of a moonlighting protein implicated in both physiological (cellular growth, migration, regulation of apoptosis) and in pathological (malignant transformation, metastasis, and cancer drug resistance) processes [6]. In the paper “Pathobiology and Therapeutic Relevance of GSK-3 in Chronic Hematological Malignancies” [7], Martelli et al. review the diverse and often still puzzling roles that the evolutionarily conserved kinase plays in the development and progression of hematological malignancies: chronic myelogenous and lymphocytic leukemias, multiple myeloma, and B-cell non-Hodgkin’s lymphomas. They also describe the arsenal of novel drugs aimed at GSK-3 activity and discuss innovative therapeutic strategies for targeting GSK-3 that could improve the outcomes of patients with blood malignancies.

Fructose 1,6-bisphosphatase (FBP) is a GSK-3-regulated multitasking protein that has become a hot topic during the last ten years, during which it became increasingly evident that FBP is not only a regulatory enzyme of glucose/glycogen synthesis but can also influence numerous non-metabolic processes. In the experimental paper “FBP2—A New Player in Regulation of Motility of Mitochondria and Stability of Microtubules in Cardiomyocytes” [8], Pietras et al. present several lines of evidence showing that the muscle isozyme of FBP (FBP2) is a regulator of microtubule-dependent dynamics of the mitochondrial network in cardiomyocytes. The chemically induced disruption of FBP2–mitochondria interaction is correlated with an increase in FBP2–Tau and FBP2–Map1B interactions, disturbance of the tubulin network and tubulin–mitochondria interaction, a marked reduction in the mitochondrial trafficking rate, and an increase in mitophagy. Therefore, the authors conclude that understanding the precise cellular mechanisms that regulate FBP2–mitochondria interactions “might be crucial for our understanding of processes that take place during physiological and pathological cardiac remodeling and during the onset of diseases that are rooted in the destabilization of microtubules and/or mitochondrial network dynamics”.

The review entitled “Calcium/Calmodulin-Stimulated Protein Kinase II (CaMKII): Different Functional Outcomes from Activation, Depending on the Cellular Microenvironment” [9] by J. Rostas and K.A. Skelding highlights the important roles of the molecular microenvironment and subcellular localization in activating different functions of CAMKII, a family of multitasking serine/threonine protein kinases. CAMKII is critical for the proper functioning of neurons and is thus implicated in neuronal pathologies such as Alzheimer’s and Parkinson’s disease, but it is also involved in the regulation of numerous processes in other cell types, including cancer cell proliferation. The authors of this review summarize the latest knowledge on the structure and regulation of CaMKII by its binding partners and explore the feasibility of developing highly selective inhibitors that target specific CaMKII-mediated functions in a given cellular context, without interfering with other processes, in different types of cells.

The post-translational modification (PTM) of a seemingly “ordinary” protein is one way to activate its non-canonical functions. Currently, over 300 types of such modifications are known [10]. In the review entitled “S-Palmitoylation of Synaptic Proteins in Neuronal Plasticity in Normal and Pathological Brains” [11], Buszka et al. describe S-palmitoylation—the attachment of a 16-carbon fatty acid to a protein—as a molecular switch necessary for the complex development of the nervous system. They present the results of the latest research aimed towards deciphering the impacts of this PTM on the properties of proteins regulating the function of synapses and synaptic plasticity in the central nervous system. They discuss the potential link between the S-palmitoylation of synaptic proteins and learning, memory, and brain disorders characterized by aberrant neuronal plasticity. They also raise questions about the regulation of the palmitoylation process which remain to be fully answered.

Another review paper entitled “UPF1—From mRNA Degradation to Human Disorders” [12] by Staszewski et al. summarizes the current knowledge on the Up-frameshift protein 1 (UPF1), an evolutionarily conserved and ubiquitous ATP-dependent RNA helicase implicated in the regulation of mRNA decay, viral genome and mRNA degradation, and the ubiquitination and formation of aggresomes. The dysfunction of this protein appears to underlie numerous human pathologies, including tumorigenesis, neurodegeneration, and viral infections. The authors comprehensively describe the structure of UPF1 in relation to the known functions of the protein and discuss its significance in human diseases. Additionally, they highlight the aspects of UPF1 function that still remain unresolved. However, despite these unanswered questions, UPF1 mutations or alterations in expression levels may be a useful markers of various diseases, and the protein itself appears to be a promising target for new therapeutic strategies.

The pleiotropic nature of proteins and the signaling pathways they create are the subject of the review entitled “Interplay of Developmental Hippo–Notch Signaling Pathways with the DNA Damage Response in Prostate Cancer” [13] by Mourkioti et al. This paper concentrates on the roles of the DNA damage response (DDR) and Hippo and Notch pathways in prostate cancer—a major cause of cancer morbidity and mortality in men worldwide. The authors recapitulate the existing knowledge on the interplay between these pathways and conclude that focusing on these pathways individually will not yield positive results in the form of new cancer treatments. Only thorough an understanding of the complex interactions between the key components of these pathways can research contribute to the identification of potential molecular targets for future therapies against prostate cancer in distinct stages of its development.

In the paper “BAG3: Nature’s Quintessential Multi-Functional Protein Functions as a Ubiquitous Intra-Cellular Glue” [14], Brenner et al. describe the ubiquitous, constitutively expressed factor BAG3 (Bcl2-associated athanogene) as a critical protein for the survival of both plants and animals. The authors postulate that this protein should be considered as a “super-multifunctional protein” and that due to its interactions with numerous proteins, BAG3 should be perceived as a “universal glue” responsible for directing its interaction partners to specific cellular compartments. They review the current literature on the roles of BAG3 in maintaining mitochondrial homeostasis and proteostasis, as well as cardiac and central nervous system pathologies and cancer. They also emphasize the needs to strengthen cooperation between scientific teams from around the world in investigating various aspects of BAG operation and to motivate new investigators to study this protein, which is so clearly involved in numerous processes in health and disease.

The phenomenon of protein multitasking has been identified in all kingdoms of life. This concept is reflected in the next three papers:

“Plant Plasma Membrane Proton Pump: One Protein with Multiple Functions” [15], by Wdowikowska et al., is an extensive review of the pleiotropic H^+^-ATPase, an integral plasma membrane protein found in plants and fungi and a key element of multiple physiological processes.

The authors focus on roles of H^+^-ATPase in reactions of plants to unfavorable environmental conditions such as pathogen attacks, non-optimal temperatures, drought, the presence of heavy metals, and high salinity. They emphasize that the phenomenon of H^+^-ATPase pleiotropy has already become a basis for the development of strategies for the improvement of crop yields, but they also discuss other potential applications of the results of research on the function of this ATPase in sustainable agriculture.

“*Francisella tularensis* Glyceraldehyde-3-Phosphate Dehydrogenase Is Relocalized during Intracellular Infection and Reveals Effect on Cytokine Gene Expression and Signaling” [16] is a research article by Pavkova et al. on GapA, the *Francisella tularensis* homologue of glyceraldehyde-3-phosphate dehydrogenase, a protein known due to its multitasking function in both animals and bacteria. The study describes the involvement of GapA in pathogenic processes at the level of host cells. The presented results suggest that both in vitro and in vivo, GapA contributes to the fully virulent manifestation of infection. Moreover, in bacteria proliferating inside primary macrophages, GapA is exposed on the surface and can be found in the host cell cytoplasm, which may suggest a role of GapA in the intracellular life cycle of *F. tularensis*. GapA appears to interact with at least two proteins within the host cell—DDX3X and S100A6—and influence ERK/MAPK signaling. These observations provide a fascinating starting point for further research on the molecular mechanisms of pathogenic processes induced by *F. tularensis.*

Ubiquitination is a protein modification responsible for directing cytosolic and nuclear proteins towards degradation by proteasome. “Ubiquitination Occurs in the Mitochondrial Matrix by Eclipsed Targeted Components of the Ubiquitination Machinery”, [17] by Zhang et al., is a research paper that provides evidence showing that in the budding yeast *Saccharomyces cerevisiae*, at least a partial form of ubiquitination machinery is present and active in the mitochondrial matrix, where it has a non-proteolytic function. Although the question of the physiological significance of this process for mitochondrial functionality remains unanswered, the results of this study present new perspectives for scientists studying ubiquitination in eukaryotic cells.

Together, the collection of papers published in this Special Issue of *Cells* clearly demonstrate the important functions played by multitasking proteins in the regulation of both physiological processes and pathological changes in cells. At the same time, the papers also lead us to realize how much remains to be discovered in this field. An in-depth understanding of the mechanisms regulating the cellular roles of multifunctional proteins can contribute not only to the creation of new effective therapies against human diseases but also to efforts for improving crop quality and the efficiency of sustainable agriculture.
